# Inactivation of Human Norovirus GII.4’s Infectivity in Fresh Oysters (*Crassostrea gigas*) through Thermal Treatment in Association with Propidium Monoazide

**DOI:** 10.3390/v16010110

**Published:** 2024-01-12

**Authors:** So Hee Kim, Pantu Kumar Roy, Eun Bi Jeon, Jin-Soo Kim, Min Soo Heu, Jung-Suck Lee, Shin Young Park

**Affiliations:** 1 Department of Seafood Science and Technology, Institute of Marine Industry, Gyeongsang National University, Tongyeong 53064, Republic of Korea; thgml9903@naver.com (S.H.K.); vetpantu88@gmail.com (P.K.R.); eunb61@naver.com (E.B.J.); jinsukim@gnu.ac.kr (J.-S.K.); 2 Department of Food and Nutrition, Gyeongsang National University, Jinju 52828, Republic of Korea; minsheu@gnu.ac.kr

**Keywords:** heat treatment, human norovirus GII.4, oyster, propidium monoazide, RT-qPCR

## Abstract

The current study investigated the effects of heat treatment (85 °C or 100 °C for 5–20 min) on human norovirus (HuNoV) GII.4’s capsid stability in fresh oysters. In addition, propidium monoazide (PMA) was used in viral samples to distinguish infectious viruses and evaluated using real-time quantitative reverse transcription polymerase chain reaction (RT-qPCR). Further, we explored the effect of the heat treatment on oyster quality (Hunter color and hardness). The titer of HuNoV for oysters significantly (*p* < 0.05) decreased to 0.39–1.32 and 0.93–2.27 log_10_ copy number/μL in the non-PMA and PMA-treated groups, respectively, after heat treatment. HuNoV in oysters not treated with PMA showed a decrease of <1.5 − log_10_, whereas in PMA-treated oysters, a decrease of >1 − log_10_ was observed after treatment at 85 °C for 10 min. Treatments for both 15 min and 20 min at 100 °C showed a >99% log_10_ reduction using PMA/RT-qPCR. In the Hunter color, an increase in heat temperature and duration was associated with a significant decrease in ‘L’ (brightness+, darkness−) and an increase in ‘a’ (redness+, greenness−) and ‘b’ (yellowness+, blueness−) (*p* < 0.05). Our findings confirmed that the hardness of oyster meat significantly increased with increasing temperature and time (*p* < 0.05). This study demonstrated that PMA/RT-qPCR was effective in distinguishing HuNoV viability in heat-treated oysters. The optimal heat treatment for oysters was 10 min at 85 °C and 5 min at 100 °C.

## 1. Introduction

Human norovirus (HuNoV) is a single-stranded positive-sense RNA virus belonging to the Caliciviridae family. A small number of virus particles (10–100) can infect an individual, and HuNoV infection is a representative cause of viral acute gastroenteritis [1,2]. HuNoV can also be transmitted by the fecal–oral route through vomiting or direct contact with an infected patient [3,4,5]. NoV is a risk factor for food poisoning worldwide and is currently the leading cause of acute gastroenteritis in the U.S., accountings for one fifth of all acute gastroenteritis cases worldwide, especially in children under 5 years of age [6]. Notably, the pooled prevalence of NoV infection among children under 5 years of age with severe gastroenteritis in the U.S. between 1990 and 2008 was 12% [7], which increased from 2015 to 2020 to 17.7% [8]. In Korea, the number of NoV outbreaks over the past 5 years was 295, and the number of patients was 4920, which is the highest incidence rate ever recorded, excluding cases of unknown cause [9]. There are seven NoV genogroups, of which GI, GII, and GIV cause infections in humans and GII, GIII, GIV, and GV cause infections in animals (cows and mice) [3,10]. Among them, the GII.4 genotype is the most frequent cause of infection, and Farahmand et al. [8] reported that the main genotypes of NoV infection from 2015 to 2020 were GII.4 and GII.3. HuNoV is environmentally resistant, with a lifespan of approximately 40 days [11]. Notably, the pathogen retains its contagiousness even after exposure to a temperature of 60 °C for 30 min. This phenomenon is particularly conspicuous in the context of bivalve mollusk shellfish, including oysters and mussels. Because these organisms tend to store waterborne contaminants in their tissue matrix, this type of food and drink is known for being more likely to become contaminated [12,13,14]. According to a European Union (EU) survey from 2018, HuNoV accounted for 20% of all outbreaks related to seafood and seafood products, including shellfish and mollusks [15].

Oysters, considered a nutritionally valuable dietary source, are characterized by their substantial glycogen content, low fat composition, and their provision of high-quality protein. Furthermore, these bivalve mollusks serve as a noteworthy reservoir of various essential nutrients, including but not limited to taurine, iron, and zinc, along with an array of vitamins and minerals [16,17]. Although oysters are nutritious, they are mainly consumed raw, which poses a high risk of food poisoning. Most oyster farms in Korea are located near the coast and are vulnerable to human pollution, including domestic wastewater and feces. In addition, limited mobility results in the accumulation of HuNoV and pathogenic bacteria in the digestive tissues of shellfish through filtration [18]. According to a European analysis, the prevalence of HuNoV in oyster farming areas was 34.5% [19]. Pathogenic bacteria accumulated in oysters can be effectively removed through shellfish purification; however, the reduction effect in regard to HuNoV is limited, and the concentration of HuNoV in oysters is 1000 times higher than that in the surrounding environment [20]. HuNoV is not completely inactivated even when oysters are consumed after heat treatment; therefore, oysters must be consumed after appropriate heat treatment to inactivate HuNoV. Moreover, other fishery products, such as fish and shellfish, should be consumed after effective heat treatment to maintain their nutritional value and quality.

Real-time reverse transcription quantitative polymerase chain reaction (RT-qPCR), a sensitive molecular biological technique, was used to test freshwater, seawater, and shellfish. This is because RT-PCR can find and measure even very small quantities of viruses. However, the inability to establish whether the RNA identified is from an infectious or non-infectious viral particle is a significant disadvantage of reverse transcriptase RT-qPCR-based technologies; further treatment is required. To get around the problems with PCR-based detection methods, nucleic acid insertion dyes have been created to stop the replication of nucleic acids in viruses that are not alive [21].

Propidium monoazide (PMA) is a nucleic acid intercalating agent that is used to treat a sample prior to RT-qPCR and helps distinguish between viable and nonviable viruses [22,23]. This dye gets inside the damaged viral capsid and binds strongly to DNA when exposed to light. This means that either the bound DNA is removed during DNA extraction or DNA goes through chemical changes that stop it from being amplified by PCR [24]. Fuentes et al. [25] also demonstrated the effects of different heat treatments on HuNoV in a study that focused on heat inactivation. They used the PMA-viability RT-qPCR assay to achieve this. This approach was employed to minimize the measurement of noninfectious viruses. Therefore, PMA with RT-qPCR is considered an appropriate method for confirming the viability of HuNoV in samples.

This study looked into what happens when heating oysters at different temperatures (85 °C or 100 °C) and for different time intervals (5, 10, 15, and 20 min). The goal was to see what effect lowering the amount of HuNoV in freshly caught oysters had. In addition, we verified the effectiveness of PMA in detecting only infectious viruses and the use of PMA for accurate results. Additionally, an assessment of oyster quality, encompassing textural attributes and Hunter color values of ‘L’ (brightness+, darkness−), ‘a’ (redness+, greenness−), and ‘b’ (yellowness+, blueness−), was concurrently conducted to determine which heat-treatment conditions were optimal, focusing on the interplay of temperature and duration pertaining to the administered heat treatments.

## 2. Materials and Methods

### 2.1. Viral Stock and Preparation

HuNoV GII. 4 was isolated from stool samples from patients with NoV-induced gastroenteritis symptoms at the Gyeonggi Institute of Health and Environment (GIHE, Gyeonggido, Republic of Korea) in 2019. After confirming the genotypes of the HuNoV GII.4, the virus was stored in a waterborne virus bank (WAVA, Seoul, Republic of Korea). HuNoV GII.4 was obtained from WAVA and prepared using a stock of 500 μL of phosphate-buffered saline (PBS, pH 7.2). Viral stocks were transported on dry ice and stored in a deep freezer at −80 °C until further use.

### 2.2. Heat Treatment of HuNoV in Oysters

Oysters were purchased from a local market in Tongyeong, Gyeongsangnam-do, Republic of Korea, and used after shell removal. The mid-gut of the oysters was collected and homogenized using a homogenizer (Daihan Scientific Co., Wonju, Republic of Korea) and divided into 3 g each in a 15 mL conical tube. For each divided sample, 10 μL of HuNoV GII.4 was inoculated and left on a clean bench for 30 min to be well absorbed by the sample. In this study, oyster homogenates were used for the heat treatment.

The sample was heat-treated at 85 °C and 100 °C in a water bath (GR150-S12, Grant Instruments Ltd., Shepreth, UK) for 5, 10, 15, and 20 min, respectively, and the treatment time was deemed to have started when the internal center temperature reached the target temperature. The exposure time was deemed to have started when the internal temperature reached the target temperature, and it took 4.3 min and 8.2 min to reach 85 °C and 100 °C, respectively. After the heat treatment, the samples were immediately immersed in ice water for 5 min to deactivate the heat. One sample was tested immediately after leaving it on a clean bench without heat treatment, which served as a control.

### 2.3. Propidium Monoazide (PMA) Treatment

To detect infective HuNoV, viral samples (700 μL) and 200 μM of PMA (Biotium, Hayward, CA, USA) were mixed before RNA extraction. This mixture was incubated in the dark at room temperature (25 °C) for 10 min to allow adequate dye penetration. Thereafter, the mixture was exposed to LED lighting at room temperature (460 nm, 40 W; Dyebio, Seongnam, Republic of Korea) for 10 min on the front and back to photoactivate the dye. In order to examine the impact of the PMA treatment on HuNoV detection, a control group was not treated with PMA.

### 2.4. Virus Isolation and RNA Extraction

A QIAamp Viral RNA Mini Kit (Qiagen, Hilden, Germany) was used to extract RNA from HuNoV according to the manufacturer’s instructions. Proteinase K was extracted according to ISO 15216-1:2017. After adding proteinase K (Sigma, St. Louis, MO, USA) to the heat-treated oyster sample, the sample was shaken in an incubator (37 °C) for 1 h and then inactivated in a water bath (60 °C) for 15 min. A centrifuge (SUPRA22K, Hanil Science Industrial Co., Gimpo, Republic of Korea) was used to spin the mixture at 5400 rpm for 10 min at 4 °C. A clear solution (approximately 3.0 mL) from the upper layer was put into a sterilized 1 mL tube. This solution was stored in a deep freezer at −80 °C, and the extracted RNA was subjected to an RT-qPCR analysis to detect and quantify HuNoV GII.4.

### 2.5. Quantitative Analysis of HuNoV Using RT-qPCR

For the quantitative analysis of HuNoV, a One-Step RT-PCR Kit (Qiagen, Hilden, Germany) was used according to the manufacturer’s instructions [14]. To amplify the genes of HuNoV GII.4, 5 µL of 5X RT-PCR buffer, 0.25 µL of RNase inhibitor (5 units/µL), 1 µL of enzyme mix (5 units/µL), 1 µL of 10 mM dNTP, 1 µL of 10 µM primer (forward and reverse), 5 µL of extracted RNA, and RNase-free water were added to a final volume of 25 µL. RT-qPCR was performed using the TP800-Thermal Cycler Device Real-Time System (TaKaRa Bio, Seoul, Republic of Korea). The primer and probe were made to fit the ORF-1 and ORF-2 overlapping areas of HuNoV GII.4 to make them more sensitive and specific. The forward and reverse primer sequences are presented in Table 1. HuNoV GII.4 was used as a positive control, and RNase-free water was used as a negative control.

### 2.6. Measurement of Hunter Color and Texture (Hardness)

Following the heat treatment, a colorimeter (UltraScan PRO, HunterLab, Reston, VA, USA) was used to measure the color of the oyster samples, which was calibrated with the original value from a standard plate (‘L’ = 98.48, ‘a’ = 0.14, and ‘b’ = 0.41). The values of ‘L’ (brightness+, darkness−), ‘a’ (redness+, greenness−), and ‘b’ (yellowness+, blueness−) were measured in triplicate for each sample.

The non-heat-treated control and heat-treated samples were analyzed using a CT3 texture analysis (Brookfield Engineering Laboratories Inc., Middleboro, MA, USA). The hardness (g/cm^2^) was assessed using the texture profile analysis. Two consecutive cycles were performed using a probe TA7 (knife-edge) and were set to reach a 70% deformation. The probe was set to a trigger force of 5.0 g at a constant speed of 5.00 mm/s.

### 2.7. Statistical Analyses

Statistical processing of the experimental results was performed for all experiments using the average value and standard deviation obtained from triplicate measurements for each sample. The statistical analysis was performed using SPSS software (version 12.0; SPSS Inc., Chicago, IL, USA), and a one-way analysis of variance (ANOVA) was conducted. We analyzed viral titers (expressed as logarithmic functions), log_10_ reduction, Hunter color, and texture using Duncan’s multiple range test [14] to compare if there were any potential differences between the means. We also used t-tests to look at how the number of viruses decreased in oyster samples treated at different temperatures and for different intervals of time compared to the non-PMA and PMA/RT-qPCR samples. Statistical significance was tested at the 5% probability level (*p* < 0.05).

## 3. Results

### 3.1. Effect of Heat Treatment on HuNoV GII.4 Reduction in Oyster Homogenates Using PMA/RT-qPCR

To analyze the efficacy of thermal treatment in eliminating HuNoV GII.4 in oyster homogenates, samples containing HuNoV GII.4 were subjected to a heat treatment at temperatures of 85 °C and 100 °C for 5–20 min. The HuNoV GII.4 initial titer of the control without thermal treatment was 3.04 log_10_ copy number/µL. The results of the non-PMA and PMA treatments of oysters inoculated with HuNoV GII.4, as analyzed by RT-qPCR, are shown in Table 2. The average titer of HuNoV GII.4 inoculated with both non-PMA-treated and PMA-treated oyster homogenates significantly decreased as the temperature and heat-treatment duration increased (*p* < 0.05). Compared to the control (3.04 log_10_ copy number/μL), the samples without PMA were reduced to 0.39 (59.26%) at 85 °C, 5 min, 0.62 (76.01%) at 85 °C, 10 min, 0.71 (80.50%) at 85 °C, 15 min, 0.83 (85.21%) at 85 °C, 20 min, 0.85 (85.87%) at 100 °C, 5 min, 0.86 (86.20%) at 100 °C, 10 min, 1.20 (93.69%) at 100 °C, 15 min, and 1.32 (95.21%) at 100 °C, 20 min log_10_ copy number/µL. No significant (*p* > 0.05) difference was observed between heat treatments at 85 °C for 10 min and 15 min Further, no significant (*p* > 0.05) difference was observed between heat treatments at 85 °C for 20 min and 5 min and at 100 °C for 5 min and 10 min. PMA treatment resulted in a reduction of 0.93 (88.25%) at 85 °C, 5 min, 1.17 (93.24%) at 85 °C, 10 min, 1.32 (95.21%) at 85 °C, 15 min, 1.42 (96.20%) at 85 °C, 20 min, 1.42 (96.20%) at 100 °C, 5 min, 1.73 (98.14%) at 100 °C, 10 min, 2.15 (99.29%) at 100 °C, 15 min, and 2.27 (99.46%) at 100 °C, 20 min log_10_ copy number/µL. No significant (*p* > 0.05) difference was observed in heat treatment at 85 °C for 15 min, 20 min, and 100 °C for 5 min. Further, no significant (*p* > 0.05) difference was observed even at 100 °C for 15 min and 20 min. Both PMA samples and those treated with PMA showed the highest decrease in HuNoV GII.4 when the temperature was high and the treatment duration was increased.

**Figure 1:**
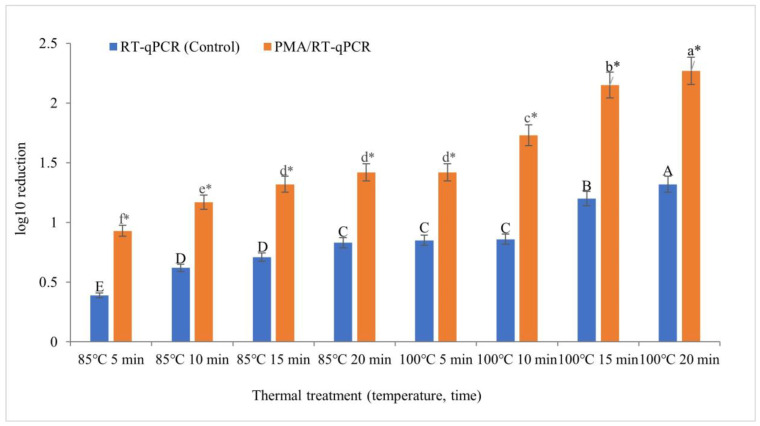
Comparison of HuNoV GII. 4 log_10_ reduction values between non-PMA and PMA-treated oyster homogenates heated at 85 °C and 100 °C for 5–20 min. At each temperature and time, the log reduction means with different letters (A–E, a–f) represent a significant difference (*p* < 0.05) between non-PMA and PMA-treated samples by Duncan’s multiple range test. Asterisks (*) also represents a significant difference (*p* < 0.05) between non-PMA and PMA-treated samples by *t*-test.

A comparison of the results between HuNoV GII.4-inoculated oyster homogenates that were not treated with PMA and those that were treated with PMA after heat treatment is shown in Figure 1. The reduction value was significantly (*p* < 0.05) higher when the PMA treatment was performed at all heat-treatment temperatures and durations. The average titer of HuNoV was confirmed to be a 0.70 log_10_ reduction (=[0.54 + 0.55 + 0.61+ 0.59 + 0.57 + 0.87 + 0.95 + 0.95 log_10_ reduction]/8) (=80% reduction) in the PMA-treated group, which was higher than that in the non-PMA-treated group. A discernible trend in PMA treatment efficacy emerged with escalating temperatures and prolonged heat exposure. Notably, the most substantial disparity was observed following heat-treatment durations of 15 and 20 min at 100 °C. Therefore, the application of PMA in conjunction with RT-qPCR subsequent to a heat treatment is an effective approach for assessing the viability of HuNoV GII.4.

### 3.2. Changes in Hunter Color and Texture of Oysters following Heat Treatment

**Figure 2:**
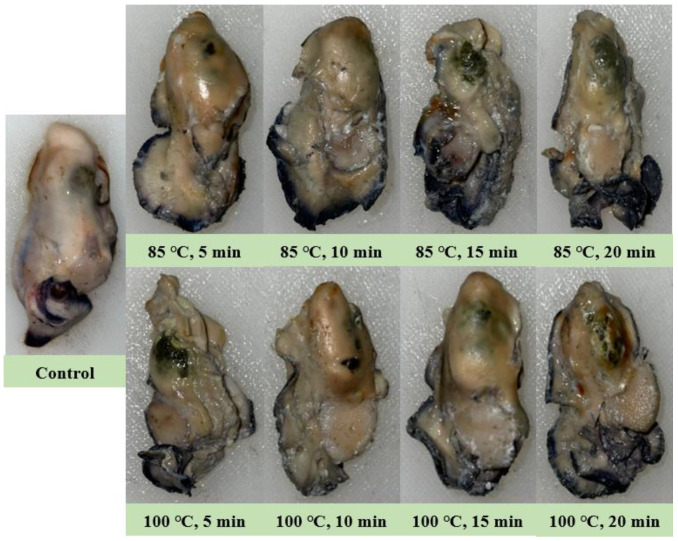
Thermal treatment by temperature and time affects the appearance of oysters.

Hunter color parameters were assessed to evaluate the differences in color after the heat treatment of the oysters. This evaluation encompassed variations attributed to both temperature and duration of treatment. The results of the Hunter color analysis are presented in Table 3. The Hunter color ‘L’ value was 50.20 for the control, and no significant difference (*p* > 0.05) was observed in the control at 85 °C after 5–10 min. However, the ‘L’ value significantly (*p* < 0.05) decreased after 15 min at 85 °C. The value was lowest when the heat treatment was performed at 100 °C for 20 min, and significantly (*p* < 0.05) decreased as the heat treatment temperature and duration increased. The ‘a’ and ‘b’ values were 0.23 and 7.07 in the control, respectively, and in contrast to the ‘L’ value, both values increased significantly (*p* < 0.05). The heat treatment induced discernible variations in the color attributes of oysters, contingent upon specific combinations of temperature and duration. The appearance of the oysters after heat treatment is shown in Figure 2. During heat treatment, oysters became dark yellow in color, and the color continued to get darker as the temperature and duration increased. Oysters heat-treated at 100 °C for 20 min had the darkest color, and the color of oyster intestines heat-treated at 100 °C for 5 min was darker than those heat-treated for 20 min at 85 °C. The external appearance and Hunter color measurements of heat-treated oysters at different temperatures for different durations showed similar results. As the color of oysters gets darker as the heat treatment temperature and duration increase, the value of ‘L’ for lightness decreases and the color becomes red and dark yellow, and the ‘a’ value for red and ‘b’ for yellow gradually increases.

The hardness of the oysters was measured after heat treatment, and the results are presented in Table 4. The hardness values of the control without heat treatment and heat treatment at 85 °C for 5 min were 114.00 and 116.50 g/cm^2^, respectively, which were significantly the lowest (*p* < 0.05). No significant (*p* > 0.05) difference was observed in oyster hardness after heat treatment at 85 °C for 15–20 min and 100 °C for 5–10 min. The hardness of oysters treated at 100 °C for 15–20 min was the same as 140.50 g/cm^2^, which was significantly (*p* < 0.05) the highest. However, no significant (*p* > 0.05) difference was observed between 15 and 20 min. Therefore, the hardness of the oysters increased as the heat treatment duration and temperature increased.

## 4. Discussion

HuNoV causes foodborne diseases worldwide through the consumption of bivalve shells and vegetables [26,27]. Considering the recurring incidence of HuNoV infections stemming from the consumption of raw oysters, a consistent pursuit of research has been aimed at devising effective control strategies. A substantial body of literature has elucidated various methods for managing the presence of norovirus in oysters. These methodologies encompass explorations into the effects of high-pressure and heat treatments on the inherent characteristics of oysters [28], electron beam irradiation, and a reduction in HuNoV GII.4 in oysters using dielectric barrier discharge (DBD) plasma [29,30]. Typically, malfunctioning septic tank systems, malfunctioning wastewater treatment plants, stormwater runoff, dumping of boat sewage waste, and vomiting overboard near shellfish areas cause HuNoV outbreaks in oysters.

Several processing and storage methodologies have been used in the food industry to enhance the intrinsic quality and value of different food products. Among these techniques, heat-based treatments including cooking and sterilization, play crucial roles. Generally, people predominantly consume shellfish, excluding oysters, after cooking them [31]. This distinction contributes to the diminished frequency of HuNoV outbreaks associated with other shellfish compared to oysters. Oysters showed the highest prevalence of HuNoV-related incidents [32]. Their prevalent consumption in raw form and occasional utilization following heat treatment, aimed at reducing the presence of foodborne microorganisms, including enteroviruses, contribute to their propensity. Heat treatment leads to a reduction in microbial spoilage, consequently extending the shelf life of food products.

In this study, the temperature and duration of exposure were set considering that heat treatment can affect oysters and that HuNoV GII.4 can be inactivated by heat treatment. The minimum temperature was set to 85 °C and the maximum temperature to 100 °C, and the exposure duration for each temperature was set at 5 min intervals up to 20 min to investigate whether HuNoV in the oysters was reduced. The thermal inactivation mechanism of viruses is related to various structural changes in the virus capsid, which depend on the temperature [33,34]. The impairment of the virus receptor binding site is confined to mild temperatures (approximately 50 °C), ensuring that the capsid maintains its function of protecting nucleic acids. However, at 60 °C or higher, the tertiary protein structure changes to facilitate thermal energy access to nucleic acid materials, resulting in their inactivation [12,27,28,29,33,34,35,36]. Ausar et al. [37] reported that norovirus-like virus particles retained their stability up to 55 °C, but the quaternary structure of the capsid changed significantly at temperatures above 60 °C. In addition, in terms of morphology, Escudero-Abarca et al. [38] reported that virus like particles of HuNoV began to get damaged after heat treatment at 70 °C for 1 min and were completely destroyed when heated at 80 °C for 1 min, as confirmed using transmission electron microscope. Furthermore, as highlighted by Park et al. [39], the production of canned oysters involved a boiling process executed at 105 °C for a duration of 6 min. The study findings indicated that HuNoV GII.4 exhibited a more pronounced reduction in viral load at a temperature of 100 °C compared to 85 °C. Park et al. [40] looked at what happened when they heated up abalone infected with murine norovirus (MNV-1). They found that heating them at 85 °C for 5 min significantly reduced MNV-1, decreasing it to 4.15 log_10_ PFU/µL compared to the control group (5.44 log_10_ PFU/µL). Similarly, Shao et al. [33] documented findings related to the heat treatment of an MVN-1-contaminated oyster homogenate. Modest reductions in MNV-1 were observed after subjecting the contaminated oyster homogenate to heat treatment at 49 °C for 12 min or at 54 °C for 6 min. However, the viral load exhibited a more substantial reduction of approximately 1.5 log10 PFU/µL following a heat exposure of 3 min at 58 °C and a notable decline of approximately 2.5 log10 PFU/µL after 2 min at 63 °C. Park et al. [41] showed that MNV-1 decreased by 3.68 log_10_ TCID_50_/mL (initial titer; 5.20 log_10_ TCID_50_/mL) when dried mussels were heat-treated at 85 °C for 10 min, and it was completely inactivated after heat treatment at 100 °C for 2–3 min. Based on the above studies, HuNoV displays a greater degree of resistance to heat treatment than MNV-1, which serves as a surrogate model for HuNoV. Although most NoV have been associated with gastrointestinal diseases in humans, some have also been identified in cattle, swine, and mice. Of these potential experimental models, MNV is the only NoV that replicates in cultures and small animals. Differences between HuNoV and MNV results are generally attributed to the influence of environmental conditions on HuNoV or its surrogate in food-affected matrices between food samples [42], which may be due to differences in experimental techniques, experimental conditions, target samples, and HuNoV strains.

Photoactive dyes, such as PMA, are known to covalently bind to DNA/RNA when exposed to strong visible light. Using this characteristic, PMA penetrates the particles of nonviable viruses under light treatment and binds to the DNA, enabling distinction between viable and nonviable viruses. Several previous studies have used these photoactive dyes in combination with RT-qPCR to differentiate between noninfectious and infectious viruses, fungi, or parasites [14]. The use of PMA/RT-qPCR as an infectivity assay relies on intact proteins to distinguish between infectious and inactivated viruses. Although photoactive dyes include PMA and ethidium monoazide (EMA), several studies [32,43] have reported that PMA is more effective than EMA. For our study, we checked if it was possible to tell the difference between infectious HuNoV particles using the PMA/RT-qPCR method. Jeong et al. [32] used RT-qPCR to confirm a decrease in HuNoV in spinach following heat treatment. When heat treatment was performed at 85 °C for 2 min, the non-dye treatment decreased by 1.01 log_10_ copy number/µL, and EMA/RT-qPCR and PMA/RT-qPCR decreased by 2.39 and 2.59 log_10_ copy number/µL, respectively. In the study conducted by Li et al. [44], a quantitative analysis of microorganisms within wastewater treatment plants (WWTP) was executed employing the PMA-qPCR technique. The results of the PMA-qPCR were strongly and positively related to the culture-based test. Roy et al. [45] reported that PMA-qPCR could detect HuNoV viability in seafood without altering its quality. The fact that these results are always the same not only shows that PMA-qPCR works, but it also supports the idea that a PMA treatment can get rid of DNA from dead viruses that are in WWTP samples. In the study by Jeon et al. [14] on the viability of HuNoV in mussels heat-treated with PMA/RT-qPCR, it was found that HuNoV levels decreased by 1.63 in the non-PMA-treated samples and 1.90 log_10_ copy number/µL in the PMA-treated samples after being heated at 90 °C for 20 min. Notably, the PMA-treated group showed a greater decrease. Our results support the notion that the PMA-treated group demonstrates a more significant reduction. In agreement with these findings, Jeon et al. [14] noted that the average titer difference between the non-PMA and PMA-treated groups for HuNoV samples was 0.27 log_10_, which is lower than the findings in the current study. The studies that were conducted and the results shown in this study make it clear that the PMA/RT-qPCR assay is a powerful and useful way to find out if HuNoV is still alive. It works well because it can stop the replication of genomic material that is not encased and viral entities that have major capsid problems.

The intestines’ chlorophyll and carotenoid pigments cause the change oyster color, and during heat treatment and storage, these pigments diffuse from the intestines to the meat to give the meat its color. Among these, carotenoid yellowing plays a leading role in discoloration [46,47]. The Hunter color and appearance after heat treatment in this study demonstrated that as the brightness decreased, the red color appeared more, and dark yellow appeared as the heat-treatment temperature and duration increased. In addition, following heat treatment, the oyster tissue becomes harder because of the outflow of moisture [48,49]. Owing to the natural variation in the traits and appearance of individual oysters, heat treatment has the potential to induce damage at the individual level. Consequently, determining the optimal temperature and duration of heat treatment becomes intricate. Photographs did not clearly show differences in oyster texture, but touching the oysters by hand (data not presented herein) and testing their texture mechanically, especially their hardness, showed that they were better after being heated for 10 min at 85 °C and 5 min at 100 °C.

## 5. Conclusions

In this study, it was demonstrated that the viability of HuNoV can be distinguished using the PMA/RT-qPCR method in oysters inoculated with HuNoV that were treated at high temperatures for a long duration (e.g., 100 °C for 20 min). Notably, the PMA agent’s ability to damage capsids affects the PMA/RT-qPCR method’s ability to tell the difference between viruses that are infectious and viruses that are not. Our findings corroborate a substantial reduction of over 1 − log_10_ following exposure to 85 °C for 10 min and a reduction exceeding 2 − log_10_ after exposure at 100 °C for 15 and 20 min, respectively. Furthermore, as the oysters underwent heat treatment at higher temperatures for extended durations, discernible shifts towards darker coloration, increased redness, and enhanced yellowness were evident. Additionally, the textural attribute of the hardness exhibited an ascending trend concomitant with higher temperatures and prolonged durations. Therefore, PMA/RT-qPCR technology could be effective in distinguishing HuNoV infectivity in oysters following heat treatment. In addition, the risk of HuNoV infection may be minimized by pursuing criteria for the thermal inactivation process for HuNoV, which can be useful for testing suitable temperatures for the high-temperature processing of food. Nevertheless, further investigation is warranted to assess the generalizability of the inactivation approach to other shellfish varieties or broader food categories.

## Figures and Tables

**Table 1:** Sequence of primer and probe for RT-qPCR.

Genotypes	Type	Component	Sequence
GII	Primer	COG1F	5′-CAR GAR BCN ATG TTY AGR TGG ATG AG-3′
		COG2R	COG2R: 5′-TCG ACG CCA TCT TCA TTC ACA-3′
	Probe	RING2	5′-TGG GAG GGC GAT CGC AAT CT-3′

**Table 2:** Effects of heat treatment on HuNoV GII.4 in oyster homogenates.

		RT-qPCR	PMA/RT-qPCR
		log_10_ Copy Number/μL	log_10_ Copy Number/μL
Control		3.04 ± 0.13 ^a^	3.04 ± 0.12 ^A^
85 °C	5 min	2.65 ± 0.03 ^b^	2.11 ± 0.04 ^B^ *
	10 min	2.42 ± 0.03 ^c^	1.87 ± 0.01 ^C^*
	15 min	2.33 ± 0.01 ^c^	1.72 ± 0.11 ^D^*
	20 min	2.21 ± 0.06 ^d^	1.62 ± 0.12 ^D^*
100 °C	5 min	2.19 ± 0.08 ^d^	1.62 ± 0.02 ^D^*
	10 min	2.18 ± 0.01 ^d^	1.31 ± 0.00 ^E^*
	15 min	1.84 ± 0.06 ^e^	0.89 ± 0.04 ^F^*
	20 min	1.72 ± 0.01 ^f^	0.77 ± 0.01 ^G^*

The letters in same row (a–f, A–G) indicate the significant differences (*p* < 0.05) for each temperature by Duncan’s multiple range test. The data represent mean values with standard deviations (three samples/treatment). Asterisk (*) represents a significant difference (*p* < 0.05) between non-PMA and PMA-treated samples by a *t*-test.

**Table 3:** Effects of heat treatment on the Hunter color of oysters.

		Hunter Color		
		‘L’	‘a’	‘b’
Control		54.41 ± 0.36 ^a^	0.23 ± 0.04 ^e^	7.07 ± 0.29 ^d^
85 °C	5 min	51.01 ± 0.41 ^b^	0.37 ± 0.13 ^e^	7.27 ± 0.19 ^d^
	10 min	50.74 ± 3.44 ^b^	0.35 ± 0.13 ^e^	7.29 ± 0.08 ^d^
	15 min	45.06 ± 1.90 ^c^	0.43 ± 0.11 ^e^	7.42 ± 0.00 ^d^
	20 min	45.26 ± 0.67 ^c^	2.27 ± 0.17 ^d^	7.51 ± 0.26 ^d^
100 °C	5 min	45.77 ± 0.58 ^c^	2.78 ± 0.22 ^c^	9.36 ± 0.91 ^c^
	10 min	44.22 ± 0.12 ^cd^	3.05 ± 0.05 ^b^	9.24 ± 0.10 ^c^
	15 min	43.94 ± 0.40 ^cd^	3.52 ± 0.17 ^a^	10.23 ± 0.08 ^b^
	20 min	41.82 ± 0.16 ^d^	3.66 ± 0.10 ^a^	11.05 ± 0.04 ^a^

The data represent means with standard deviations (three samples/treatment). The letters in the same row (a–e) indicate the significant differences (*p* < 0.05) for each temperature by Duncan’s multiple range test. ‘L’ values = Lightness, ‘a’ values = redness+, ‘b’ values = yellowness+.

**Table 4:** Effects of heat treatment on the texture of oysters.

Texture		Hardness (g/cm^2^)
Control		114.00 ± 0.00 ^b^
85 °C	5 min	116.50 ± 19.10 ^b^
	10 min	126.50 ± 14.85 ^a,b^
	15 min	127.00 ± 11.31 ^a,b^
	20 min	127.50 ± 3.54 ^a,b^
100 °C	5 min	135.00 ± 22.63 ^a,b^
	10 min	134.00 ± 0.00 ^a,b^
	15 min	140.50 ± 4.95 ^a^
	20 min	140.50 ± 9.19 ^a^

The data represent means with standard deviations (three samples/treatment). The letters in the same row (a,b) indicate the significant differences (*p* < 0.05) for each temperature by Duncan’s multiple range test.

## Data Availability

Data are contained within the article. The data presented in this study are available on request from the corresponding author.

## References

[1] Das O., Lekshmi M., Kumar S., Nayak B.B. (2020). Incidence of norovirus in tropical seafood harbouring fecal indicator bacteria. Mar. Pollut. Bull..

[2] Patel M.M., Hall A.J., Vinjé J., Parashar U.D. (2009). Noroviruses: A comprehensive review. J. Clin. Virol..

[3] Graaf M.D., Beek J.V., Koopmans M.P.G. (2016). Human norovirus transmission and evolution in a changing world. Nat. Rev. Microbiol..

[4] Wikswo M.E., Hall A.J. (2012). Outbreaks of acute gastroenteritis transmitted by person-to-person contact–United States, 2009–2010. MMWR Surveill. Summ..

[5] Li D., Zhao M.Y., Tan T.H.M. (2021). What makes a foodborne virus: Comparing coronaviruses with human noroviruses. Curr. Opin. Food Sci..

[6] Atmar R.L., Ramani S., Estes M.K. (2018). Human noroviruses: Recent advances in a 50-year history. Curr. Opin. Infect. Dis..

[7] Patel M.M., Widdowson M.A., Glass R.I., Akazawa K., Vinjé J., Parashar U.D. (2008). Systematic literature review of role of noroviruses in sporadic gastroenteritis. Emerg. Infect. Dis..

[8] Farahmand M., Moghoofei M., Dorost A., Shoja Z., Ghorbani S., Kiani S.J., Khales P., Esteghamati A., Sayyahfar S., Jafarzadeh M. (2021). Global prevalence and genotypes distribution of norovirus infection in children with gastroenteritis: A meta-analysis on 6 years of research from 2015 to 2020. Rev. Med. Virol..

[9] Ministry of Food and Drug Safety (MFDS) (2023). Food Poisoning Statistics of Norovirus. https://www.foodsafetykorea.go.kr/portal/healthyfoodlife/foodPoisoningStat.do?menu_no=4425&menu_grp=MENU_NEW02.

[10] Zheng D.P., Ando T., Fankhauser R.L., Beard R.S., Glass R.I., Monroe S.S. (2006). Norovirus classification and proposed strain nomenclature. Virology.

[11] Yang M., Zhao F., Tong L., Wang S., Zhou D. (2022). Contamination, bioaccumulation mechanism, detection, and control of human norovirus in bivalve shellfish: A review. Crit. Rev. Food Sci. Nutr..

[12] Heaton J.C., Jones K. (2008). Microbial contamination of fruit and vegetables and the behaviour of enteropathogens in the phyllosphere: A review. J. Appl. Microbiol..

[13] Duizer E., Schwab K.J., Neill F.H., Atmar R.L., Koopman M.P.G., Estes M.K. (2004). Laboratory efforts to cultivate noroviruses. J. Gen. Virol..

[14] Jeon E.B., Choi M.-S., Kim J.Y., Ha K.S., Kwon J.Y., Jeong S.H., Lee H.J., Jung Y.J., Ha J.H., Park S.Y. (2020). Characterizing the effects of thermal treatment on human norovirus GII.4 viability using propidium monoazide combined with RT-qPCR and quality assessments in mussels. Food Control.

[15] Fusco G., Anastasio A., Kingsley D.H., Amoroso M.G., Pepe T., Fratamico P.M., Cioffi B., Rossi R., La Rosa G., Boccia F. (2019). Detection of hepatitis a virus and other enteric viruses in shellfish collected in the gulf of naples, Italy. Int. J. Environ. Res. Public Health.

[16] Son K.T., Shim K.B., Lim C.W., Yoon N.Y., Seo J.H., Jeong S.G., Yeong W.Y., Cho Y.J. (2014). Relationship of pH, Glycogen, Soluble Protein, and Turbidity between freshness of raw oyster Crassostrea gigas. Korean J. Fish. Aquat. Sci..

[17] Bartlett J.K., Maher W.A., Purss M.B. (2018). Near infra-red spectroscopy quantitative modelling of bivalve protein, lipid and glycogen composition using single-species versus multi-species calibration and validation sets. Spectronchim. Acta A Mol. Biomol. Spectrosc..

[18] Lowther J.A., Gustar N.E., Powell A.L., Hartnell R.E., Less D.N. (2012). Two-year systematic study to assess norovirus contamination in oysters from commercial harvesting areas in the United Kingdom. Appl. Environ. Microbiol..

[19] Romalde J.L., Rivadulla E., Varela M.F., Barja J.L. (2018). An overview of 20 years of studies on the prevalence of human enteric viruses in shellfish from Galicia, Spain. J. Appl. Microbiol..

[20] Martinez-Albores A., Lopez-Santamarina A., Rodriguez J.A., Ibarra I.S., Mondragón A.D.C., Miranda J.M., Lamas A., Cepeda A. (2020). Complementary methods to improve the depuration of bivalves: A review. Foods.

[21] Frankenhuyzen J.K.V., Trevors J.T., Lee H., Flemming C.A., Habash M.B. (2011). Molecular pathogen detection in biosolids with a focus on quantitative PCR using propidium monoazide for viable cell enumeration. J. Microbiol. Methods.

[22] Slimani S., Robyns A., Jarraud S., Molmeret M., Dusserre E., Mazure C., Facon J.P., Lina G., Etienne J., Ginevra C. (2012). Evaluation of propidium monoazide (PMA) treatment directly on membrane filter for the enumeration of viable but non cultivable *Legionella* by qPCR. J. Microbiol. Methods.

[23] Parshionikar S., Laseke I., Fout G.S. (2010). Use of propidium monoazide in reverse transcriptase PCR to distinguish between infectious and noninfectious enteric viruses in water samples. Appl. Environ. Microbiol..

[24] Karim M.R., Fout G.S., Johnson C.H., White K.M., Parshionikar S.U. (2015). Propidium monoazide reverse transcriptase PCR and RT-qPCR for detecting infectious enterovirus and norovirus. J. Virol. Methods.

[25] Fuentes C., Pérez-Rodríguez F.J., Sabrià A., Beguiristain N., Pintó R.M., Guix S., Bosch A. (2021). Inactivation of hepatitis a virus and human norovirus in clams subjected to heat treatment. Front. Microbiol..

[26] Kingsley D.H., Vincent E.M., Meade G.K., Watson C.L., Fan X. (2014). Inactivation of human norovirus using chemical sanitizers. Int. J. Food Microbiol..

[27] Berger C.N., Sodha S.V., Shaw R.K., Griffin P.M., Pink D., Hand P., Frankel G. (2010). Fresh fruit and vegetables as vehicles for the transmission of human pathogens. Environ. Microbiol..

[28] Romero C.M., Kelly A.L., Kerry J.P. (2007). Effects of high-pressure and heat treatments on physical and biochemical characteristics of oysters (*Crassostrea gigas*). Innov. Food Sci. Emerg. Technol..

[29] Kim S.E., Park S.Y., Rui M.-L., Ha S.-D. (2017). Effects of electron beam irradiation on murine norovirus-1 in abalone (*Haliotis discus hannai*) meat and viscera. LWT Food Sci. Technol..

[30] Ahlfeld B., Li Y., Boulaaba A., Binder A., Schotte U., Zimmermann J.L., Morfill G., Klein G. (2015). Inactivation of a foodborne norovirus outbreak strain with nonthermal atmospheric pressure plasma. MBio.

[31] Bhat Z.F., Morton J.D., Bekhit A.E.D.A., Kumar S., Bhat H.F. (2021). Thermal processing implications on the digestibility of meat, fish and seafood proteins. Compr. Rev. Food Sci. Food Saf..

[32] Jeong M.I., Park S.Y., Ha S.-D. (2017). Thermal inactivation of human norovirus on spinach using propidium or ethidium monoazide combined with real-time quantitative reverse transcription-poSSslymerase chain reaction. Food Control.

[33] Shao L., Chen H., Hicks D., Wu C. (2018). Thermal inactivation of human norovirus surrogates in oyster homogenate. Int. J. Food Microbiol..

[34] Song H., Li J., Shi S., Yan L., Zhuang H., Li K. (2010). Thermal stability and inactivation of hepatitis C virus grown in cell culture. Virology.

[35] Bozkurt H., D’Souza D.H., Davidson P.M. (2015). Thermal inactivation of foodborne enteric viruses and their viral surrogates in foods. J. Food Prot..

[36] Croci L., Suffredini E., Di Pasquale S., Cozzi L. (2012). Detection of norovirus and feline Calicivirus in spiked molluscs subjected to heat treatments. Food Control.

[37] Ausar S.F., Foubert T.R., Hudson M.H., Vedvick T.S., Middaugh C.R. (2006). Conformational stability and disassembly of Norwalk virus-like particles. Effect of pH and temperature. J. Biol. Chem..

[38] Escudero-Abarca B.I., Rawsthorne H., Goulter R.M., Suh S.H., Jaykus L.A. (2014). Molecular methods used to estimate thermal inactivation of a prototype human norovirus: More heat resistant than previously believed?. Food Microbiol..

[39] Park J.-S., Park D.-H., Kong C.-S., Lee Y.-M., Lee J.-D., Park J.-H., Kim J.-G. (2018). Processing and characteristics of canned seasoned boiled oyster *Crassostrea gigas* and canned seasoned roasted oyster *Crassostrea gigas*. Korean J. Fish. Aquat. Sci..

[40] Park S.Y., Bae S.-C., Ha S.-D. (2015). Heat inactivation of a norovirus surrogate in cell culture lysate, abalone meat, and abalone viscera. Food Environ. Virol..

[41] Park S.Y., Kim S.-H., Ju I.-S., Cho J.-I., Ha S.D. (2014). Thermal inactivation of murine norovirus-1 in suspension and in dried mussels (*Mytilus edulis*). J. Food Saf..

[42] Topping J.R., Schnerr H., Haines J., Scott M., Carter M.J., Willcocks M.M., Bellamy K., Brown D.W., Gray J.J., Gallimore C.I. (2009). Temperature inactivation of feline *Calicivirus* vaccine strain FCV F-9 in comparison with human noroviruses using an RNA exposure assay and reverse transcribed quantitative real-time polymerase chain reaction—A novel method for predicting virus infectivity. J. Virol. Methods.

[43] Coudray-Meunier C., Fraisse A., Martin-Latil S., Guillier L., Perelle S. (2013). Discrimination of infectious hepatitis A virus and rotavirus by combining dyes and surfactants with RT-qPCR. BMC Microbiol..

[44] Li D., Tong T., Zeng S., Lin Y., Wu S., He M. (2014). Quantification of viable bacteria in wastewater treatment plants by using propidium monoazide combined with quantitative PCR (PMA-qPCR). J. Environ. Sci..

[45] Roy P.K., Jeon E.B., Kim J.Y., Park S.Y. (2023). Application of High-Pressure Processing (or High Hydrostatic Pressure) for the Inactivation of Human Norovirus in Korean Traditionally Preserved Raw Crab. Viruses.

[46] Chen F., Zhang M., Fan K., Mujumdar A.S. (2022). Non-thermal technology and heating technology for fresh food cooking in the central kitchen processing: A review. Food Rev. Int..

[47] Park W.-J., Jwa M.-K., Hyun S.-H., Lim S.B., Song D.-J. (2006). Microbial and quality changes during storage of raw oyster treated with high hydrostatic pressure. J. Korean Soc. Food Sci. Nutr..

[48] Nath K.G., Pandiselvam R., Sunil C.K. (2023). High-pressure processing: Effect on textural properties of food-A review. J. Food Eng..

[49] Kong C.-S., Yun J.-U., Oh D.-H., Park J.-Y., Kang J.-Y., Oh K.-S. (2009). Effects of high temperature sterilization on qualities characteristics of the canned boiled oyster. J. Agric. Life Sci..

